# Bortezomib-based consolidation or maintenance therapy for multiple myeloma: a meta-analysis

**DOI:** 10.1038/s41408-020-0298-1

**Published:** 2020-03-06

**Authors:** Shijia Zhang, Amit A. Kulkarni, Beibei Xu, Haitao Chu, Taxiarchis Kourelis, Ronald S. Go, Michael L. Wang, Veronika Bachanova, Yucai Wang

**Affiliations:** 1 Division of Hematology, Oncology and Transplantation, Department of Medicine, University of Minnesota, Minneapolis, MN USA; 2 Medical School, University of Minnesota, Minneapolis, MN USA; 3 Division of Biostatistics, School of Public Health, University of Minnesota, Minneapolis, MN USA; 4 Division of Hematology, Mayo Clinic, Rochester, MN USA; 5 Department of Lymphoma and Myeloma, The University of Texas MD Anderson Cancer Center, Houston, TX USA

**Keywords:** Myeloma, Myeloma

## Abstract

Bortezomib-based regimens are widely used as induction therapy for multiple myeloma (MM). Unlike lenalidomide, the role of bortezomib in consolidation and maintenance therapy for MM is less clear. We performed a meta-analysis to evaluate the impact of bortezomib-based consolidation and maintenance therapy on survival outcomes and adverse events. PubMed, Web of Science, Embase databases, and major conference proceedings were searched for randomized controlled trials (RCTs) of bortezomib-based regimens as consolidation or maintenance therapy for MM. Ten RCTs enrolling 3147 patients were included in the meta-analysis. Bortezomib-based regimens were compared with regimens without bortezomib or observation. The meta-analysis suggested that bortezomib-based maintenance therapy improved progression-free survival (PFS; hazard ratio [HR] = 0.72, 95% CI 0.55–0.95, *P* = 0.02) and overall survival (OS; HR = 0.71, 95% CI 0.58–0.87, *P* = 0.001). Bortezomib-based consolidation therapy improved PFS (HR = 0.77, 95% CI 0.68–0.88, *P* < 0.001) but not OS (HR = 0.98, 95% CI 0.78–1.24, *P* = 0.87). Bortezomib-based consolidation/maintenance therapy led to a trend toward increased risk of grade ≥ 3 neurologic symptoms, gastrointestinal symptoms, and fatigue. More research is warranted to further assess the role of bortezomib-based consolidation and maintenance therapy for multiple myeloma.

## Introduction

Multiple myeloma (MM) is a clonal plasma cell neoplasm that is associated with significant morbidity and mortality. It is the second most common hematologic malignancy, accounting for about 1% of all cancers^1^. Better understanding of the disease’s pathophysiology has led to recent advances in therapy and improved patient outcomes dramatically. The initial treatment of newly diagnosed MM patients who are transplant-eligible is induction chemotherapy with a triple-drug regimen followed by high-dose chemotherapy and autologous stem cell transplantation (ASCT). For transplant-ineligible patients, triplet or doublet drug combinations are typically recommended for induction therapy^2^. Despite therapeutic advancements and the availability of novel drugs, disease relapse is inevitable for the majority of patients after the initial treatment. Therefore, a large portion of patients are given consolidation or maintenance therapy with the intent to prolong progression-free survival (PFS) and overall survival (OS). Consolidation therapy is a short course of treatment to deepen the response to the initial therapy. Maintenance therapy aims to extend the period of disease quiescence with a longer course of a less-intensive regimen^3^.

At the time of analysis, lenalidomide was the only Food and Drug Administration (FDA)-approved drug in the United States (US) for maintenance therapy after ASCT in MM. Although generally well-tolerated, lenalidomide is associated with increased risk for neutropenia, thrombocytopenia, anemia, infections, thromboembolism, and second primary cancers^4,5^. Bortezomib is a first-in-class proteasome inhibitor that can lead to cell-cycle arrest and apoptosis^6^. Bortezomib-based regimens are widely used as induction therapy for MM^7–9^. Bortezomib has been used off label for consolidation or maintenance therapy after the initial treatment of MM, particularly for those with high-risk disease^10^. Unlike lenalidomide, the role of bortezomib in the consolidation or maintenance setting is less clear. Therefore, we conducted this meta-analysis of randomized controlled trials (RCTs) to examine the efficacy and safety of bortezomib-based regimens as consolidation or maintenance therapy in MM following induction therapy with or without ASCT. Bortezomib-based regimens were compared with regimens without bortezomib or observation.

## Methods

### Search strategy and selection criteria

PubMed, Web of Science, and Embase databases were searched for RCTs of bortezomib-based regimens (either single-agent or combination) as consolidation or maintenance therapy for MM through 31 August 2019. The keywords used for the literature search were “myeloma,” “bortezomib,” and “consolidation OR maintenance OR continuous therapy.” We also searched for abstracts presented at the *American Society of Clinical Oncology* or *American Society of Hematology* annual conferences. The references of relevant articles were manually searched to identify any additional eligible clinical trials. Studies eligible for inclusion met all the following criteria: (1) RCTs; (2) participants with MM; (3) studies with an intervention group given a bortezomib-containing regimen for consolidation/maintenance vs. a control group given either a bortezomib-free regimen or no consolidation/maintenance therapy; (4) studies reporting PFS and/or OS; and (5) studies published in English. Studies in both the transplantation setting and non-transplantation setting were included. Two investigators (S.Z. and Y.W.) independently conducted the literature search and screened the clinical trials. Discrepancies were resolved through consensus.

### Study outcomes and data extraction

Efficacy outcomes included PFS and/or OS. Safety outcomes included treatment-related grade 3 or higher adverse events. For each included trial, we extracted the name of the first author, year of publication or conference presentation, study design, ASCT status, treatment setting (consolidation vs. maintenance), and study arm. We also retrieved the hazard ratio (HR) with 95% confidence interval (CI) of survival outcomes (PFS and/or OS) and grade ≥ 3 adverse event data. For studies that did not report HRs for survival outcomes, the authors were contacted for additional information.

### Statistical analysis

Data synthesis was performed according to the guidelines for meta-analyses^11^. Pooled HRs of survival outcomes with 95% CI were calculated with the inverse variance method^12^ and pooled risk ratio (RR) of dichotomous safety data with 95% CI were computed with the Mantel–Haenszel and DerSimonian–Laird methods^13,14^. Study heterogeneity was assessed using the *I*^2^ statistic^15^. Forest plots were constructed for each meta-analysis to examine and display study-level data. The *I*^2^ statistic was used to describe the percentage of the variation across studies that is due to between-study differences rather than chance. Common cutoff points for low (*I*^2^ = 25%), moderate (*I*^2^ = 50%), and high degrees of heterogeneity (*I*^2^ = 75% or higher) were used^15^. Sensitivity analysis was performed by repeating the meta-analysis, excluding each individual study one at a time. Publication bias was evaluated by the funnel plot and Begg’s rank correlation test^16^. Analysis was conducted with MedCalc 16.2 (MedCalc Software, Ostend, Belgium) and Comprehensive Meta Analysis V3 (Biostat, Englewood, New Jersey, USA), using the random-effects model^14^. All statistical analyses were two-sided. A *P*-value < 0.05 was considered statistically significant, except for the heterogeneity analysis as mentioned above.

## Results

### Search results

**Fig. 1:**
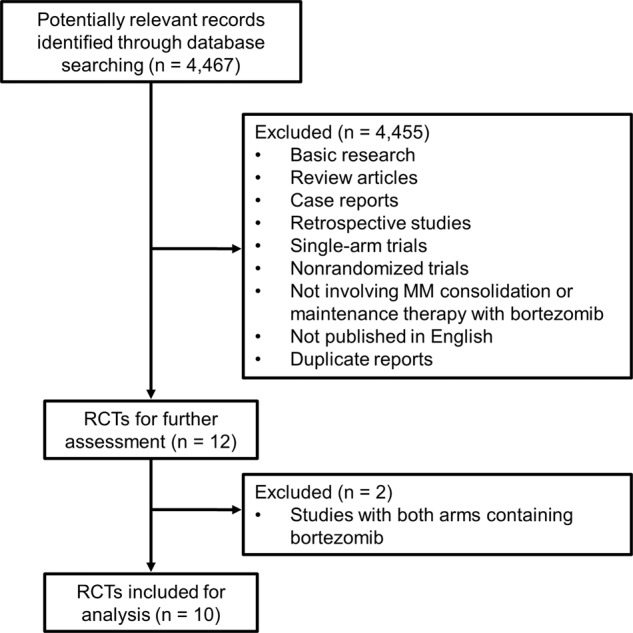
Flow diagram of search results. RCT randomized controlled trial.

We identified 4467 references from the initial database search and kept 12 RCTs for further review after initial screening. Basic research studies, review articles, case reports, retrospective studies, single-arm trials, non-randomized trials, studies not involving MM consolidation or maintenance therapy with bortezomib, duplicate reports, or publications not in English were excluded. Two RCTs of MM consolidation or maintenance therapy were excluded, as both study arms contained bortezomib^17,18^. Ten RCTs (in nine publications) were included in the final meta-analysis (Fig. 1).

### Characteristics of included trials

Of the ten included RCTs^19–27^, there were nine phase III studies and one phase II study included in the analysis. Bortezomib-based regimens were administered as consolidation therapy in seven RCTs^19–22,25,27^ and as maintenance therapy in three RCTs^23,24,26^, following the initial therapy. Only one RCT was conducted in the non-transplantation setting^24^. The outcomes of the MMY3012 trial (NCT00416273) and MMY3013 trial (NCT00416208) were published as a pre-specified single dataset analysis on the combined data^21^. They were considered as one study in our meta-analysis. A total of 3147 patients were included: 1506 participants received bortezomib-based regimens, and 1641 received non-bortezomib-based regimens or no consolidation/maintenance (Table 1).

**Table 1:** Description of included studies.

Study	Setting of treatment	Initial therapy regimen	Consolidation/maintenance regimen	Patient No.
Stadtmauer et al.^19^^a^	Consolidation	Heterogeneous regimens, ~80% pts received bor	Bor 1.3 mg/m^2^ i.v. days 1, 4, 8, and 11 + len 15 mg/d days 1 to 14 + dex 40 mg/d days 1, 8, and 15; 21-day cycle, 4 cycles	254
			No consolidation	257
Horvath et al.^20^	Consolidation	Bor s.c. + cyclophosphamide + dex	Bor 1.3 mg/m^2^ s.c. every 2 weeks, 32 weeks + thal 100 mg/d, 1 year + prednisolone 50 mg on alternate days	103
			Thal 100 mg/d, up to 1 year + prednisolone 50 mg on alternate days	100
Einsele et al.^21^^b^	Consolidation	Heterogeneous regimens, 50% pts received bor	Bor 1.6 mg/m^2^ i.v. days 1, 8, 15, and 22; 35-day cycle, 4 cycles	177
			Observation	180
Sezer et al.^22^^c^	Consolidation	Heterogeneous regimens, 68% pts received bor	Bor 1.6 mg/m^2^ i.v. days 1, 8, 15, and 22; 35-day cycle, 4 cycles	51
			Observation	53
Mellqvist et al.^25^^b^	Consolidation	Heterogeneous regimens, no pts received bor	Bor 1.3 mg/m^2^ i.v. days 1, 4, 8, and 11, 21-day cycle, 2 cycles, then days 1, 8, and 15, 28-day cycle, 4 cycles	187
			Observation	183
Cavo et al.^27^	Consolidation	Bor + thal + dex	Bor 1.3 mg/m^2^ i.v. days 1, 8, 15, and 22 + thal 100 mg/d + dex 40 mg/d days 1, 2, 8, 9, 15, 16, 22, and 23; 35-day cycle, 2 cycles	160
		Thal + dex	Thal 100 mg/d + dex 40 mg/d days 1, 2, 8, 9, 15, 16, 22, and 23; 35-day cycle, 2 cycles	161
Rosinol et al.^23^^b^	Maintenance	Heterogeneous regimens, 34% pts received bor	Bor 1.3 mg/m^2^ i.v. days 1, 4, 8, and 11 every 3 months + thal 100 mg/d, up to 3 years	91
			Thal 100 mg/d, up to 3 years	88
			Alfa2-IFN starting at 1.5 MU (could be increased to 3 MU) s.c. 3 times per week, up to 3 years	92
Palumbo et al.^24^^d^	Maintenance	Bor + mel + prednisone + thal	Bor 1.3 mg/m^2^ i.v. every 15 days + thal 50 mg/d, up to 2 years	254
		Bor + mel + prednisone	Observation	257
Sonneveld et al.^26,35,36^^b^	Maintenance	Bor + doxorubicin + dex	Bor 1.3 mg/m^2^ i.v. every 2 weeks, up to 2 years	229
		Vincristine + doxorubicin + dex	Thal 50 mg/d, up to 2 years	270

*Alfa2-IFN* interferon alfa-2b, *ASCT* autologous stem cell transplantation, *bor* bortezomib, *dex* dexamethasone, *i.v*. intravenous, *len* lenalidomide, *MU* million units, *pts* patients, *s.c*. subcutaneous, *thal* thalidomide.

^a^Only the two relevant arms were shown in the table.

^b^The authors provided additional updated data (personal communication) that were used in this meta-analysis.

^c^Open-label phase II trial; all others were open-label phase III trials.

^d^No ASCT; all others included ASCT.

### Survival outcomes for bortezomib-based consolidation therapy

**Fig. 2:**
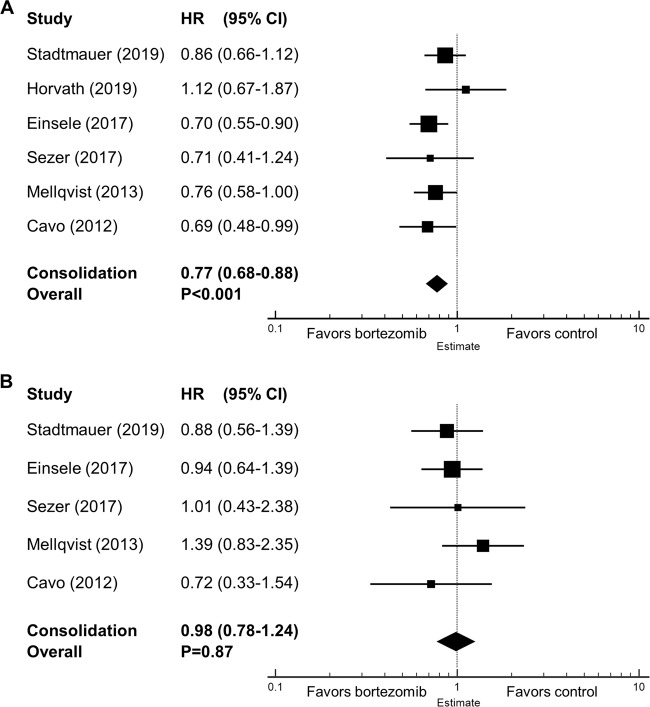
Forest plots of hazard ratios (HRs) in the consolidation setting. (**a**) HRs for progression-free survival and (**b**) HRs for overall survival of bortezomib-based regimen vs. control. HRs for each trial are represented by squares, where the size of the square represents the weight of the trial in the meta-analysis and the horizontal line crossing the square represents the 95% confidence interval (CI). The diamonds represent the overall summary HR estimates and 95% CIs.

Pooled data from the included trials showed that bortezomib-based consolidation therapy significantly improved PFS (HR = 0.77, 95% CI 0.68–0.88, *P* < 0.001), but not OS (HR = 0.98, 95% CI 0.78–1.24, *P* = 0.87) as compared with no consolidation or regimens without bortezomib (Fig. 2). Given that only one arm in Cavo’s study^27^ received bortezomib in induction and consolidation, we excluded this trial and repeated the meta-analysis to better assess the impact of bortezomib-containing regimen in the consolidation phase. This did not change the overall result and demonstrated improved PFS (HR = 0.79, 95% CI 0.68–0.90, *P* < 0.001), but not OS (HR = 1.01, 95% CI 0.79–1.29, *P* = 0.92). Of note, the OS data of the VCAT trial^20^ were not available for the meta-analysis.

### Survival outcomes for bortezomib-based maintenance therapy

**Fig. 3:**
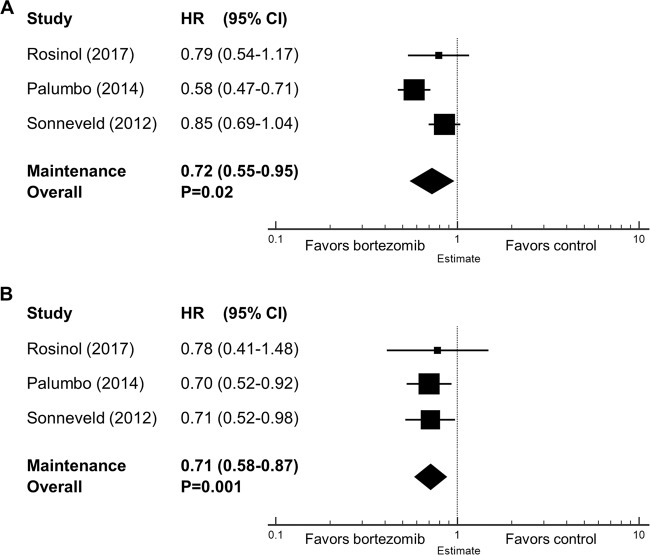
Forest plots of hazard ratios (HRs) in the maintenance setting. (**a**) HRs for progression-free survival and (**b**) HRs for overall survival of bortezomib-based regimen vs. control. HRs for each trial are represented by squares, where the size of the square represents the weight of the trial in the meta-analysis and the horizontal line crossing the square represents the 95% confidence interval (CI). The diamonds represent the overall summary HR estimates and 95% CIs.

Bortezomib-based maintenance therapy significantly increased PFS (HR = 0.72, 95% CI 0.55–0.95, *P* = 0.02) and OS (HR = 0.71, 95% CI 0.58–0.87, *P* = 0.001) as compared with no maintenance or regimens without bortezomib (Fig. 3). To assess the role of bortezomib-based maintenance therapy specifically in patients after ASCT, we excluded the trial involving transplant-ineligible patients^24^ and performed the meta-analysis again, which showed improved OS (HR = 0.72, 95% CI 0.54–0.96, *P* = 0.025) and a clear trend toward improved PFS (HR = 0.84, 95% CI 0.699–1.001, *P* = 0.052).

Of note, one trial^23^ had three maintenance arms: bortezomib + thalidomide (VT) vs. thalidomide (T) vs. alfa-2b interferon (IFN). The pooled HRs shown above were based on the HRs between VT and T, as interferon is rarely used for MM in the current era. We also calculated the pooled HRs using the HRs between VT and IFN, and this did not change the overall conclusion (data not shown).

The meta-analysis was conducted in both MedCalc 16.2 and Comprehensive Meta Analysis V3 with same results.

### Adverse events

Regarding safety, we calculated the pooled RRs for grade 3 or higher adverse events. Bortezomib-based consolidation/maintenance therapy induced a trend toward increased risk of grade ≥ 3 neurologic symptoms (RR = 1.59, 95% CI 0.94–2.69, *P* = 0.08), gastrointestinal symptoms (RR = 1.66, 95% CI 0.71–3.88, *P* = 0.24), and fatigue (RR = 2.10, 95% CI 0.83–5.30, *P* = 0.12) as compared with no consolidation/maintenance or regimens without bortezomib. However, these findings did not reach statistical significance (Table 2). Of note, subcutaneous bortezomib was used in only one study (in both the initial therapy and consolidation therapy)^20^ based on available data in these publications.

**Table 2:** Meta-analysis of grade ≥ 3 adverse events.

Grade ≥ 3 AE	No. of RCT	Events in bor-based arm	Events in control arm	RR	95% CI	*P*
Neurologic	8	125/1457	86/1587	1.59	0.94–2.69	0.08
Gastrointestinal	6	114/1127	95/1178	1.66	0.71–3.88	0.24
Fatigue	3	17/665	8/708	2.10	0.83–5.30	0.12

*AE* adverse events, *bor* bortezomib, *CI* confidence interval, *RR* risk ratio

### Heterogeneity analysis

We evaluated the heterogeneity of the studies using the *I*^2^ statistic. There was no clear evidence of statistical heterogeneity for the consolidation PFS (*I*^2^ = 0%), consolidation OS (*I*^2^ = 0%), or maintenance OS (*I*^2^ = 0%) data. There was moderate heterogeneity in the maintenance PFS (*I*^2^ = 71.47%) data. We repeated the meta-analysis for the survival outcomes using the fixed-effects model and the results did not change the overall conclusions of this study (data not shown).

### Sensitivity analysis

Sensitivity analysis was carried out by removing one study at a time and repeating the meta-analysis to evaluate the stability of the results. The pooled HRs ranged from 0.75 to 0.80 for PFS and from 0.90 to 1.02 for OS in the consolidation setting. The pooled HRs ranged from 0.65 to 0.84 for PFS and from 0.70 to 0.72 for OS in the maintenance setting. The analysis showed that the results were overall stable.

### Publication bias

Publication bias was assessed by formal tests^16^. The *P*-value for consolidation PFS and OS from the Begg’s rank correlation test was 0.71 and 1.0, respectively. The *P*-value for maintenance PFS and OS from the Begg’s rank correlation test was 1.0 and 0.30, respectively. The funnel plots are shown in Supplementary Fig. 1. These results demonstrate that there is no indication of significant publication bias among the included RCTs.

## Discussion

Despite the introduction of several novel drugs and combination regimens in MM in recent years, maintaining remission after induction therapy is challenging and remains an unmet need. Consolidation and maintenance therapies are the strategies to prolong remission and survival in these patients. Our meta-analysis demonstrated that bortezomib-based maintenance significantly prolonged PFS and OS in MM patients after induction therapy in the absence or presence of ASCT. However, consolidation therapy with bortezomib-containing regimens only improved PFS but not OS. To our knowledge, our study is the first comprehensive meta-analysis evaluating the benefits and risks of bortezomib-based consolidation and maintenance therapy in patients with MM. By pooling data from multiple studies (even though the majority of which did not show conflicting results), our study provides a higher level of evidence regarding the role of bortezomib in consolidation and maintenance therapy for MM.

### Bortezomib-based consolidation therapy

Consolidation therapy for MM is given as a short course of chemotherapy after the initial therapy, particularly in patients who have undergone ASCT. We identified seven RCTs (published in six articles)^19–22,25,27^ comparing consolidation with bortezomib-based regimens vs. non-bortezomib-based regimens or no consolidation. Consolidation was given after ASCT in all seven trials. The pooled analysis suggested delayed disease progression with bortezomib-based consolidation. However, the published RCTs evaluating the impact of consolidation with bortezomib-containing regimens on OS have reported different results. Studies led by Cavo et al.^27^, Einsele et al.^21^, and Stadtmauer et al.^19^ showed that consolidation with bortezomib-containing regimens led to a trend toward improved OS. In contrast, Mellqvist et al.^25^ showed the opposite. The contradictory effects on OS shown in Mellqvist’s study might be due to the fact that all patients were bortezomib-naive before consolidation and more patients in the control group received bortezomib-containing intensive therapy after first relapse (48/183 vs. 19/187)^25^. The study by Sezer et al.^22^ revealed similar effects on OS in the bortezomib arm compared with observation (HR = 1.01). Of note, none of these studies was statistically significant in terms of OS. The pooled HR of these studies was 0.98, indicating no OS advantage with bortezomib-based consolidation. The BMT CTN 0702 trial was the only study to properly isolate the effects of consolidation from maintenance. In this well-designed trial, consolidation with four cycles of bortezomib, lenalidomide, and dexamethasone prior to lenalidomide maintenance did not significantly improve OS^19^. One of the advantages of consolidation therapy is that treatment can be completed in a relatively short period of time. This could potentially decrease the risk of toxicities compared with maintenance therapy, in which more doses of bortezomib are given. However, the value of consolidation chemotherapy as a concept remains to be proven in MM and it needs studies such as the BMT CTN 0702 trial^19^, wherein the effect of consolidation is isolated. Consolidation therapy for MM is not the preferred approach in our clinical practice given the lack of OS benefit.

### Bortezomib-based maintenance therapy

Maintenance therapy refers to a course of low-dose chemotherapy over a long period of time for patients with MM after induction therapy ± ASCT. The duration of maintenance therapy is usually 2–3 years or until disease progression, relapse, or unacceptable toxicity. Three RCTs evaluated the role of maintenance therapy with bortezomib-containing regimens in MM. Two studies were conducted in the transplant setting^23,26^ and one in transplant-ineligible patients^24^. The study by Palumbo et al.^24^ revealed that maintenance with bortezomib + thalidomide was superior to observation in terms of PFS and OS. Sonneveld et al.^26^ found that bortezomib led to significantly prolonged OS, but not PFS, as compared with thalidomide in the maintenance setting. Rosinol et al.^23^ compared three maintenance regimens: bortezomib + thalidomide vs. thalidomide vs. alfa-2b interferon and showed that bortezomib + thalidomide resulted in a trend toward improved PFS and OS. Our meta-analysis suggested that maintenance with bortezomib-based regimens prolonged both PFS and OS (pooled HR = 0.72 and 0.71, respectively).

It is not entirely clear why OS was improved with bortezomib-based regimens in the maintenance setting, but not in the consolidation setting. One can hypothesize that continuous suppression of myeloma cells with a bortezomib-containing regimen over a long period of time is needed to have a positive effect on OS. The flip side is that more exposure to bortezomib can potentially cause more side effects. It raises the question whether bortezomib-based maintenance for high-risk patients should be preferred over bortezomib-based consolidation given the lack of OS benefit with consolidation therapy. In our practice, we prefer maintenance therapy with bortezomib 1.3 mg/m^2^ every 2 weeks for certain high-risk patients. We typically treat patients for at least 2 years or till disease progression. The duration of treatment should also be based on toxicities, tolerance, and cost. Future studies are warranted to answer these questions.

### Adverse events

Peripheral neuropathy is one of the dose-limiting toxicities of bortezomib and dose modification or discontinuation is often required in clinical practice. Our meta-analysis showed a trend of increased risk of grade ≥ 3 neurologic adverse events with bortezomib-based consolidation/maintenance. Bortezomib is also known to cause gastrointestinal symptoms, fatigue, and thrombocytopenia, etc. Although there is a trend toward increased grade ≥ 3 adverse events, our meta-analysis showed that bortezomib did not significantly increase the rate of these events. This may be partially explained by the fact that not all studies reported these adverse events, resulting in a relatively small patient number in the meta-analysis. Also, the route of administration (intravenous vs. subcutaneous) and the side effects resulting from induction therapy, pre-transplant high-dose chemotherapy, ASCT, and other medications (i.e., thalidomide) in the control arm during consolidation/maintenance might have confounded the results. Nevertheless, patients should be closely monitored for side effects during treatment.

### Bortezomib vs. lenalidomide

Immunomodulatory drugs have been extensively studied in consolidation and maintenance therapy for MM. Posttransplant maintenance with lenalidomide is commonly utilized as it is the only US Food and Drug Administration-approved drug for this indication. In a meta-analysis of RCTs published in the *Journal of Clinical Oncology*, McCarthy et al.^28^ demonstrated both PFS benefit and OS benefit with lenalidomide maintenance after ASCT. However, its use has been limited by side effects including second primary neoplasms, less activity in high-risk disease, and high out-of-pocket costs to patients. In fact, some patients choose not to take oral lenalidomide for maintenance because of the financial burden its use imposes and prefer a non-oral medication. Here we show that bortezomib-based maintenance therapy improves both PFS and OS. Furthermore, bortezomib does not need dose adjustment in patients with renal impairment. Therefore, bortezomib may be an alternative option for certain patients.

Our colleagues at Mayo Clinic suggest a risk-adapted approach after initial therapy: lenalidomide maintenance is recommended for standard-risk disease, whereas bortezomib or carfilzomib-based maintenance is reserved for high-risk disease^10^. This approach is based on the finding that certain high-risk features, such as translocation between chromosomes 4 and 14 [t(4;14)], can be overcome by bortezomib^29,30^, and those high-risk patients would benefit more from a bortezomib-based regimen^31,32^. The role of bortezomib in patients with chromosome 17p deletion [del(17p)] is controversial. Although some studies suggested that bortezomib may negate the poor prognosis conferred by del(17p)^7,33^, this was not observed in a large study of 507 patients by Avet-Loiseau et al.^30^. We attempted to perform a subgroup analysis of high-risk patients; however, not enough data were presented in these published studies for such a meta-analysis. Nevertheless, high-quality evidence is needed to support these recommendations and further studies may identify which patient groups could derive the most benefit from bortezomib-based or lenalidomide-based therapy for consolidation and maintenance.

### Limitations

Our study has a few limitations. Frist, the study is limited by a relatively small number of clinical trials, particularly for trials of maintenance therapy and trials in the non-transplantation setting. Second, abstracted data from published RCTs instead of individual patient data were used for the meta-analysis. Therefore, a subgroup analysis of high-risk patients could not be performed. Third, the RCTs had apparent heterogeneities in the patient population, study design, induction therapy regimen, bortezomib dose, schedule, and other factors. Some trials used bortezomib in the induction therapy and continued to use a bortezomib-based regimen for consolidation/maintenance. A second randomization would help delineate the role of a bortezomib-based regimen in the setting of consolidation/maintenance therapy. For example, Rosinol et al.^23^ did a second randomization before starting maintenance therapy. However, in the studies by Cavo et al.^27^ and Sonneveld et al.^26^, only one arm received bortezomib in the induction phase and consolidation/maintenance phase, and no randomization was performed before entering consolidation/maintenance, making it difficult to isolate the impact of bortezomib-containing regimens in consolidation/maintenance. In the consolidation setting, we repeated the meta-analysis by excluding Cavo’s trial and found similar outcomes. A similar analysis in the maintenance setting could not be performed because of the small number of studies. These considerations can potentially confound our results. However, it is unlikely that the survival differences seen are due to induction therapy, because clinical trials that solely evaluated differences in induction regimens have seldom shown an effect on OS. For example, the IFM 2005–01 trial compared the efficacy of bortezomib + dexamethasone vs. vincristine + doxorubicin + dexamethasone as induction therapy before ASCT, but it failed to demonstrate a significant OS benefit^34^. Thus, the effect on OS shown in the meta-analysis is likely due to maintenance therapy. More clinical trials in this area are needed to confirm our results. Given the heterogeneity of the included trials, we decided to use the random-effects model to conduct the meta-analysis of survival outcomes. Caution should be used when interpreting results from this meta-analysis.

## Conclusion

This meta-analysis showed that consolidation therapy with bortezomib-containing regimens only improved PFS but not OS in patients with MM. Despite the limitations of this analysis, we have some suggestion of the benefit of bortezomib-based maintenance therapy, particularly in high-risk patients. Further research is warranted to assess the role of bortezomib in maintenance therapy for MM. Given that different patient groups may respond differently to a specific regimen, we believe a risk-adapted approach should be used in future studies to tailor consolidation and maintenance therapy based on disease risks, regimens used in induction therapy, and minimal residual disease status.

## Supplementary information

Supplementary Figure 1

Supplementary Figure 1 Legend
