# Supplementary figures and images for: Bortezomib-based consolidation or maintenance therapy for multiple myeloma: a meta-analysis
Source: Blood Cancer J. 2020 Mar 6;10(3):33. doi: 10.1038/s41408-020-0298-1 (PMC7060191; doi:10.1038/s41408-020-0298-1)

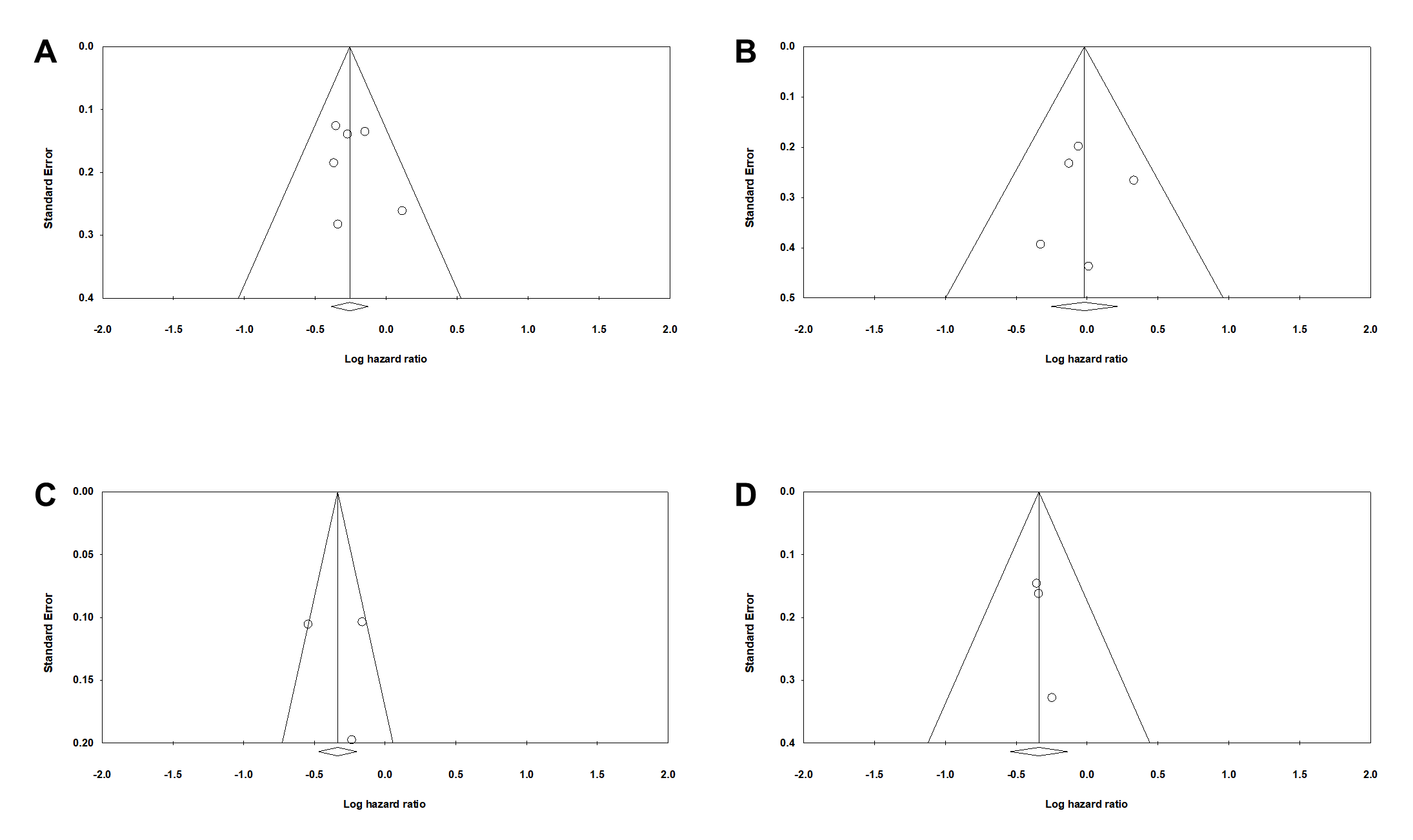

Supplement: Supplementary file 1 — Supplementary Figure 1 [file 41408_2020_298_MOESM1_ESM.tif]
