# Supplementary material for: Bortezomib-based consolidation or maintenance therapy for multiple myeloma: a meta-analysis
Source: Blood Cancer J. 2020 Mar 6;10(3):33. doi: 10.1038/s41408-020-0298-1 (PMC7060191; doi:10.1038/s41408-020-0298-1)
Supplement: Supplementary file 2 — Supplementary Figure 1 Legend [file 41408_2020_298_MOESM2_ESM.docx]

Supplementary Figure 1.

Funnel plots of publication bias analysis of studies on (A) consolidation progression-free survival, (B) consolidation overall survival, (C) maintenance progression-free survival, and (D) maintenance overall survival of bortezomib-based regimen versus control.
